# Improving Valproate Prescribing Practices in Women of Childbearing Age: An Audit Cycle in a Psychiatric Inpatient Setting in Qatar

**DOI:** 10.1192/bjo.2025.10542

**Published:** 2025-06-20

**Authors:** Ahmad Alater, Zidan Masoudi, Sazgar Hamad, Majid Al Abdulla, Yousaf Iqbal

**Affiliations:** Hamad Medical Corporation, Doha, Qatar

## Abstract

**Aims:** Prescribing valproate to women of childbearing age in psychiatric settings requires a nuanced approach due to its teratogenicity. The Medicines and Healthcare Products Regulatory Agency (MHRA) and the National Institute for Health and Care Excellence (NICE) guidelines emphasize the importance of avoiding valproate in this population, considering alternative medications, discussing benefits, side effects, and teratogenicity, conducting pregnancy testing before initiation, and ensuring the use of highly effective contraception. This study aimed to evaluate current prescribing practices against MHRA and NICE guidelines within Hamad Medical Corporation (HMC) psychiatric inpatient units.

**Methods:** An initial audit was conducted between 25/10/2022 and 25/10/2023, followed by a re-audit between 10/11/2023 and 10/11/2024. The audit involved a retrospective review of electronic health records of all female patients of childbearing age (16–49) admitted to the psychiatry hospital and prescribed valproate for a psychiatric indication. Data were collected using a proforma, and the audit was approved by the HMC Audit Committee.

**Results:** During the first audit period, 32 patients were prescribed valproate. Of these, only 1 patient (3%) had documented discussion about teratogenicity, 7 patients (21%) about benefits, and 3 patients (9%) about side effects. 21 patients (65.6%) underwent pregnancy testing before prescription. However, none of the patients received documented education about highly effective contraception.

In response to these findings, results were widely disseminated within the department, and educational sessions were conducted for doctors and pharmacists. Additionally, the need to develop national guidelines was emphasized to ensure safer prescribing practices. Noteworthy, during the re-audit phase, there was an expansion in bed capacity for female patients.

The re-audit showed a reduction in valproate prescribing to 21 patients. Documented discussions on teratogenicity increased to 10 patients (47.6%), while 5 patients (23.8%) had discussions about benefits and 14 patients (66.7%) about side effects. Pregnancy testing before prescribing improved to 19 patients (90%). Additionally, 8 patients (38.1%) received documented education on effective contraception.

**Conclusion:** The re-audit demonstrated significant improvement in pregnancy testing, and noticeable progress in other aspects, though further work is required. This study underscores the importance of educational sessions and interdisciplinary collaboration among doctors and pharmacists to enhance prescribing practices. Towards sustained improvement, systematic changes are needed, including shifting clinicians’ perceptions of valproate prescribing, developing local guidelines, and introducing strict governance measures. This audit has served as a catalyst for the development and implementation of national guidelines and has led to the initiation of a quality improvement project.

